# Improvement of loperamide-induced slow transit constipation by *Bifidobacterium bifidum* G9-1 is mediated by the correction of butyrate production and neurotransmitter profile due to improvement in dysbiosis

**DOI:** 10.1371/journal.pone.0248584

**Published:** 2021-03-22

**Authors:** Yutaka Makizaki, Taiki Uemoto, Haruka Yokota, Miyuki Yamamoto, Yoshiki Tanaka, Hiroshi Ohno

**Affiliations:** R&D Center, Biofermin Pharmaceutical Co., Ltd., Kobe, Japan; University of Louisville School of Medicine, UNITED STATES

## Abstract

A treatment option for constipation that improves the quality of life is needed since available laxatives do not effectively improve the quality of life in patients with constipation. A significant association between gut dysbiosis and constipation is recognized, suggesting that probiotics may be an important option for management of constipation. The underlying mechanism by which probiotics improve constipation remains unclear. In this study, we aimed to evaluate the effects of the probiotic *Bifidobacterium bifidum* G9-1 (BBG9-1) on loperamide-induced delayed colonic transit constipation and to elucidate its mechanism of action. First, the effect of BBG9-1 was evaluated in a rat model of constipation induced by subcutaneous administration of loperamide. BBG9-1 improved constipation parameters (number of feces, fecal water content, and fecal hardness) in constipated rats. Next, the relationship of organic acids and neurotransmitters to gut microbiota was investigated. BBG9-1 improved dysbiosis and prevented a decrease in butyric acid concentration in the gut, increased serum serotonin, and suppressed an increase in dopamine and a decrease in acetylcholine in serum. Further, an increase in the expression level of tryptophan hydroxylase 1, a 5-HT-synthetizing enzyme, was observed. These results suggest that BBG9-1 improves dysbiosis, which results in an increase in organic acids and improvement of neurotransmission. These actions may increase intestinal mobility, finally leading to alleviating constipation. The probiotic BBG9-1 may, therefore, be a potential option for the treatment of constipation.

## Introduction

Chronic constipation is a common functional gastrointestinal disorder that affects approximately 16% of the population, although prevalence varies by country and age [1, 2]. This illness is classified as slow transit constipation (STC), normal transit constipation, or pelvic floor dysfunction/defecatory disorder [3]. STC, defined as significantly prolonged colonic transit time due to decreased colonic motility, results in a decrease in frequency and quantity of stool. The condition occurs most commonly in the elderly, who are more likely to have impaired intestinal function. Constipated patients have a significantly lower quality of life (QOL) than healthy individuals [4]. Even with the administration of currently available laxatives, QOL remains poor in constipated patients due to the unsatisfactory therapeutic effect outcomes and side effects [5]. Thus, a treatment option to improve the QOL of patients with constipation is needed.

Gut dysbiosis is reported in patients with chronic constipation [6], suggesting a significant association between gut dysbiosis and constipation. Probiotics can balance gut microbiota [7] and alleviate constipation by shortening colonic transit time and increasing fecal water content and other fecal parameters [8–10]. Also, probiotics can improve the QOL of patients with constipation [11]. Given these findings probiotics may be an effective treatment modality for constipation. However, the mechanism underlying probiotic-induced changes in gut microbiota that alleviate the condition remains unclear.

The autonomic nervous system regulates intestinal motility. When the parasympathetic nervous system is excited, acetylcholine (Ach) is released to activate gastrointestinal motility. Among the neurotransmitters, serotonin (5‐hydroxytryptamine, 5‐HT) and dopamine (DA) play a role in Ach release: when the 5-HT receptors are stimulated, the extent of Ach release increases. On the other hand, when DA receptors are stimulated, the extent of Ach release reduces, resulting in decreased gastrointestinal motility. It has been demonstrated that colonic 5-HT levels are decreased in patients with constipation [12] and that there exists a close relationship between 5-HT and gut microbiota [13]. Therefore, we believe that the gut microbiota may be significantly involved in the autonomic regulation of intestinal motility.

In this study, we aimed to confirm the effect of the probiotic *Bifidobacterium bifidum* G9-1 (BBG9-1) on constipation in a rat model of loperamide-induced STC and to understand its mechanism of action.

## Materials and methods

### Bacterial strain and culture conditions

Bacterial strain BBG9-1 was obtained from Biofermin Pharmaceutical Co., Ltd. (Kobe, Japan). BBG9-1 was anaerobically cultured for 18 h at 37°C in GAM broth (Nissui Pharmaceutical Co., Ltd., Tokyo, Japan) supplemented with 0.7% glucose and 0.1% Tween-80. The bacteria were washed twice with phosphate-buffered saline, and the pellets obtained after low-speed centrifugation were stored at −80°C until use.

### Animals

Sixty seven-week-old male Sprague Dawley rats were purchased from Japan SLC (Hamamatsu, Japan) and acclimated to the laboratory conditions for one week before use in the study. The sample size was calculated based on the results of the preliminary study. The number of animals used in each study was shown in the legend of appropriate Figures. The animals were housed individually in stainless steel five-unit cages (CL-02036, W 755 × D 210 × H 170 mm; CLEA Japan, Inc., Tokyo, Japan) under specific pathogen-free conditions at room temperature (22°C ± 3°C) and a humidity of 55% ± 15%, with a 12-h light/dark cycle (07:00–19:00). In addition, a CE-2 powdered diet (CLEA Japan) was provided *ad libitum* as a standard diet and tap water was freely available from a water supply bottle. The experiments were conducted according to the Biofermin Pharmaceutical animal experiment guidelines after approval from the Biofermin Pharmaceutical Animal Experiment Committee (approval number: 133–006).

### Induction of constipation and study design

The male Sprague Dawley rats were randomly assigned to one of three groups: normal, loperamide-induced constipation, and BBG9-1-treated loperamide-induced constipation groups (hereinafter referred to as the Normal group, Lop group, and BBG9-1 group, respectively). Rats in Lop and BBG9-1 groups were subcutaneously injected with 5.0 mg of loperamide hydrochloride (Sigma, St. Louis, MO, USA), twice a daily, for 4 consecutive days to induce constipation. Rats in the Normal group were subcutaneously injected with physiological saline. Rats in the BBG9-1 group also received BBG9-1 by gavage at a dose of 1.0×10^10^ CFU, three times a day (9:00, 13:00, 17:00), for 4 consecutive days; rats in the Normal and Lop groups received PBS by gavage instead of BBG9-1. On the day following the completion of treatments with loperamide and BBG9-1, whole blood (7 mL) was collected under isoflurane anesthesia. The contents of the cecum and feces in the colon were removed, rapidly frozen, and stored at −80°C until analyzed.

### Measurement of fecal number and fecal weight

The number and weight of feces were determined for all feces excreted between 9:00 am each day and 9:00 am the following day during the treatment period from day 0 to day 4.

### Measurement of fecal water content

Fresh feces were collected by gently compressing the abdomens, immediately weighed, and dried at 90°C for 24 h. Fecal water content was determined as the difference in fecal weight before and after drying.

### Measurement of fecal hardness

Fresh feces were collected by gently pressing the abdomen, and the fecal hardness was measured using a food hardness testing unit (DSV-50N-M4, Imada, Japan).

### Measurement of residual fecal numbers in the colon

After drawing whole blood, the intestinal segment from the cecum to the anus was removed, and the number of fecal pellets remaining in the colon was counted.

### Gastrointestinal transit time (GITT)

Rats were given 1 mL of carmine (Fujifilm Wako, Japan) by gavage, prepared by suspending 3 g in 50 ml of 0.5% carboxymethylcellulose, on the last day of loperamide administration (Day 3). GITT was measured as the time from oral gavage to the appearance of dye-stained feces.

### Measurement of neurotransmitters in serum

A laparotomy was performed under isoflurane anesthesia to collect blood from the abdominal aorta. Collected blood was allowed to clot overnight at room temperature and then centrifuged at 2,000 g for 15 min to separate the serum.

Serum 5‐HT levels were determined using a Serotonin Research ELISA Kit (BA E-5900; ImmuSmol SAS, Pessac, France), and serum DA levels were determined using a Dopamine Research ELISA Kit (BS E-5300; ImmuSmol SAS, Pessac, France). Both neurotransmitters were extracted from serum samples, acylated, and assayed according to the manufacturer’s instructions.

Ach was assayed in filtered serum samples using an Acetylcholine Assay Kit (STA-603; Cell Biolabs, Inc. USA) according to the manufacturer’s instructions.

### Measurement of organic acid concentrations in cecal contents

Concentrations of organic acids (succinic acid, lactic acid, acetic acid, propionic acid, n-butyric acid, and n-valeric acid) in cecal contents were determined by high-performance liquid chromatography. Briefly, an appropriate cecal contents was accurately weighed into a tube, suspended in MilliQ water, and heat-treated (85°C, 20 min). After homogenizing with beads (0.1 mm in diameter), the sample was centrifuged (14,000 g, 10 min). The supernatant was filtered through 0.45 μm membrane filters and used for analysis.

High-performance liquid chromatography was performed using a Shim-pack SCR-102 (H) column (300 mm × 8 mm ID, Shimadzu, Kyoto, Japan), an electrical conductivity detector (CDD-10A, Shimadzu, Kyoto, Japan), and an Organic Acid Analytical System (Shimadzu, Kyoto, Japan). The eluent containing 5 mmol/L p-toluenesulfonic acid was delivered at a flow rate of 0.8 ml/min at 50°C.

### Quantitative real-time PCR of tryptophan hydroxylase-1 (Tph-1) in colon tissue

Colon tissue, 50 to 100 mg, was weighed, and RNA was extracted with Trizol^®^ reagent (Ambion, Life Technologies Japan Ltd, Tokyo, Japan). Extracted RNA was reverse transcribed to cDNA using PrimeScript^®^ RT Master Mix (Takara Bio Inc., Shiga, Japan). Quantitative real-time PCR was performed with cDNA using a Quant Studio™ 3 Real-Time PCR System (Applied Biosystems™, Thermo Fisher Scientific K K., Tokyo, Japan) and PowerUp™ SYBR™ Green Master Mix (Applied Biosystems™, Thermo Fisher Scientific K K., Tokyo, Japan). PCR was run in Fast mode using the protocol for the PowerUp™ SYBR™ Green Master Mix: hold step at 50°C for 2 min and at 95°C for 2 min followed by thermal denaturation and extension at 95°C for 1 s and 60°C for 30 s for 40 cycles. Primer sequences for the Tph-1 gene were obtained from the PrimerBank website (http://pga.mgh.harvard.edu/primerbank/) as ID209862984c3: Tph-1-forward: 5’-CCATCTTCCGAGAGCTAAACAAA-3’ and Tph-1-reverse: 5’-TCTTCCCGATAGCCACAGTATT-3’. The following primers were used as the endogenous control: GAPDH-forward: 5’-TGCACCACCAACTGCTTAG-3’ and GAPDH-reverse: 5’-GATGCAGGGATGATGTTC-3’ [14]. Expression levels of the Tph-1 in colon tissue were quantified by the ΔΔCt method and are expressed relative to expression in Normal group tissues.

### Fecal microbiological analyses

We used 16S rRNA gene sequencing for comprehensive gut microbiota analysis. DNA was extracted from feces using the bead-phenol method [15]. The V3-V4 region of the 16S rRNA gene was sequenced on the Miseq platform with a Miseq reagent kit version 2 (500 cycles, Illumina) according to the method of Fadrosh *et al* [16]. The Raw read data obtained from the Miseq platform were processed on the QIIME pipeline [17] as follows: reads were merged using the Fastq-join program and quality filtered (QV ≥ 25) using USEARCH software version 6.1. Chimeric sequences were removed from data that passed quality filtering to obtain reliable data for microbiota analysis. A total of 5,000 reads per specimen were randomly extracted and clustered into operational taxonomic units using the USEARCH algorithm with a 97% similarity threshold. The representative sequence of each operational taxonomic units was assigned taxonomy by homology research using the UCLUST tool, so that each read was identified to the genus level. Dissimilarity indices (Bray–Curtis distances) were calculated for gut microbiota composition in each specimen with the vegdist function (vegan package) of R statistical software and used for principal coordinates analysis (PCoA).

### Statistical analysis

The experimental results are expressed as mean ± standard error. Homogeneity of variance was assessed using Bartlett’s test, and significance of differences was tested using the Steel-Dwass test for unequal variances and Tukey-Kramer test for equal variances. The correlation between fecal water content and hardness was assessed by the Pearson’ correlation coefficient.

## Results

### Effect of BBG9-1 on fecal parameters

**Fig 1:**
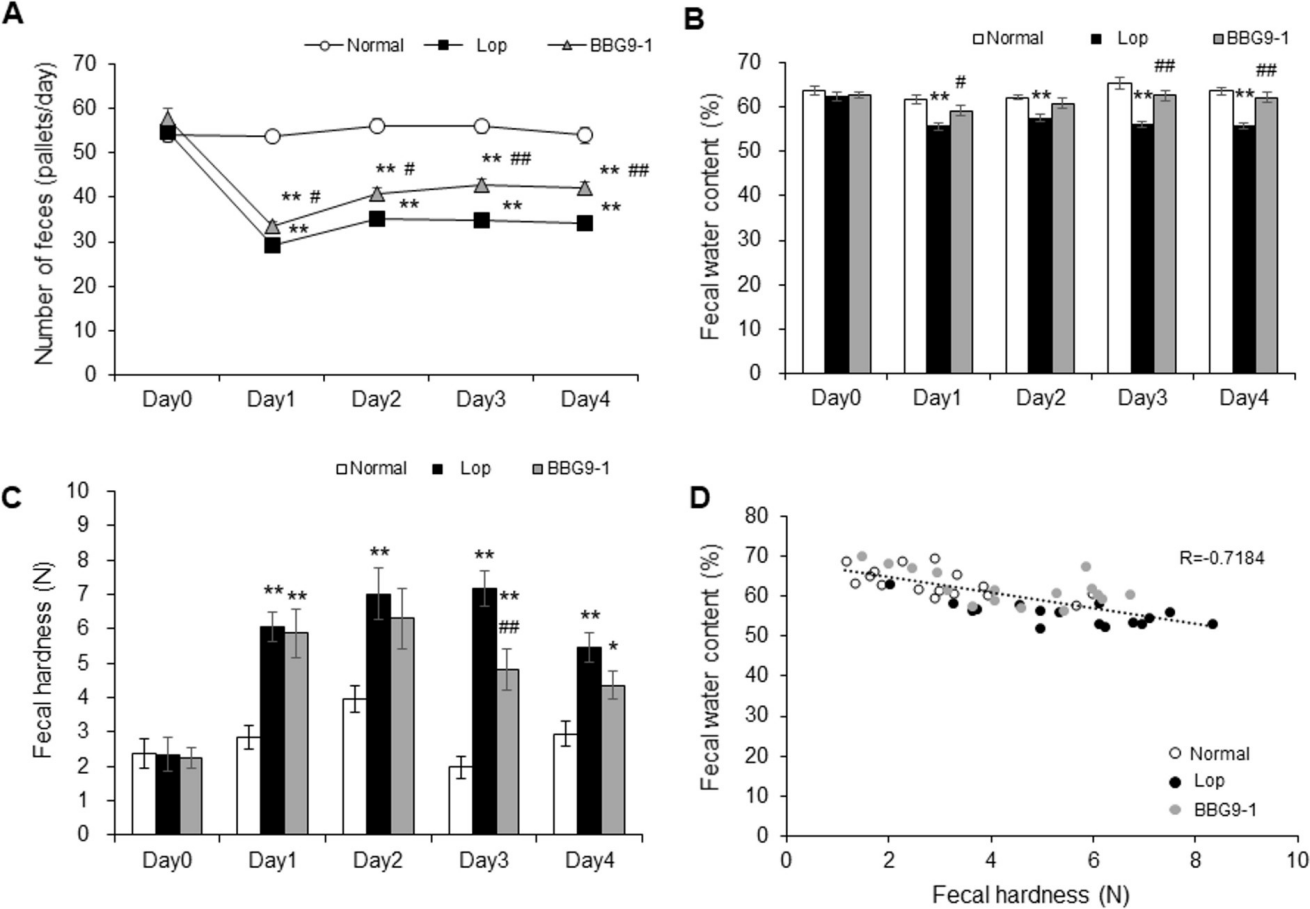
Change in fecal properties in rats with loperamide-induced constipation. (A) Number of feces, (B) Fecal water content, and (C) Fecal hardness were measured every experimental day. (D) Correlation between fecal water content and hardness was assessed at day 4. Values are means ± standard error (SE) of 16 animals. **, p < 0.01 vs. Normal; # and ##, p < 0.05 and 0.01 vs. Lop.

Numbers and weights of fecal pellets, fecal water content, and fecal hardness were significantly decreased at day 1 and thereafter in the Lop group, compared with the same parameters in the Normal group (Fig 1A–1C). Compared with the Lop group, the BBG9-1 group had significantly higher number of feces on days 1–4 (Fig 1A) and significantly higher fecal water content on days 3 and 4 (Fig 1B) and significantly reduced fecal hardness at day 3 (Fig 1C). A negative correlation between the fecal water content and hardness was found (Pearson correlation coefficient, −0.7184; Fig 1D), indicating that fecal hardness reduced with increasing fecal water content.

### Effect of BBG9-1 on residual fecal number in the colon

**Fig 2:**
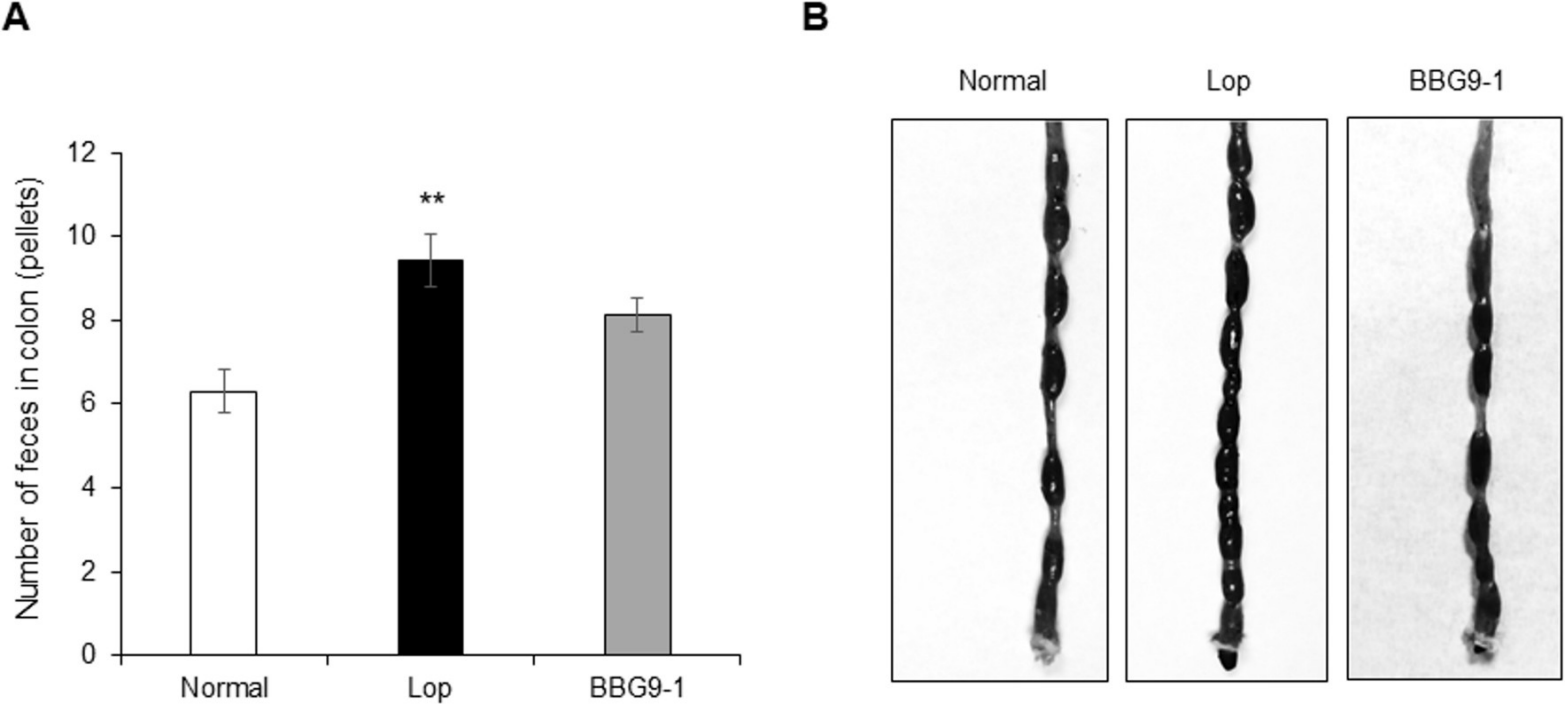
Effect of BBG9-1 on colon contents. Loperamide-induced rats were dissected on day 4, and (A) the number of feces in the colon was counted. (B) Representative examples of the colon of animals in each group. Values are means ± SE of 16 animals. **, p < 0.01 vs. Normal.

The number of fecal pellets in the colon was significantly higher in animals in the Lop group than in the Normal group. BBG9-1 group had lower numbers of residual feces than the Lop group (Fig 2).

### Effect of BBG9-1 on GITT

**Fig 3:**
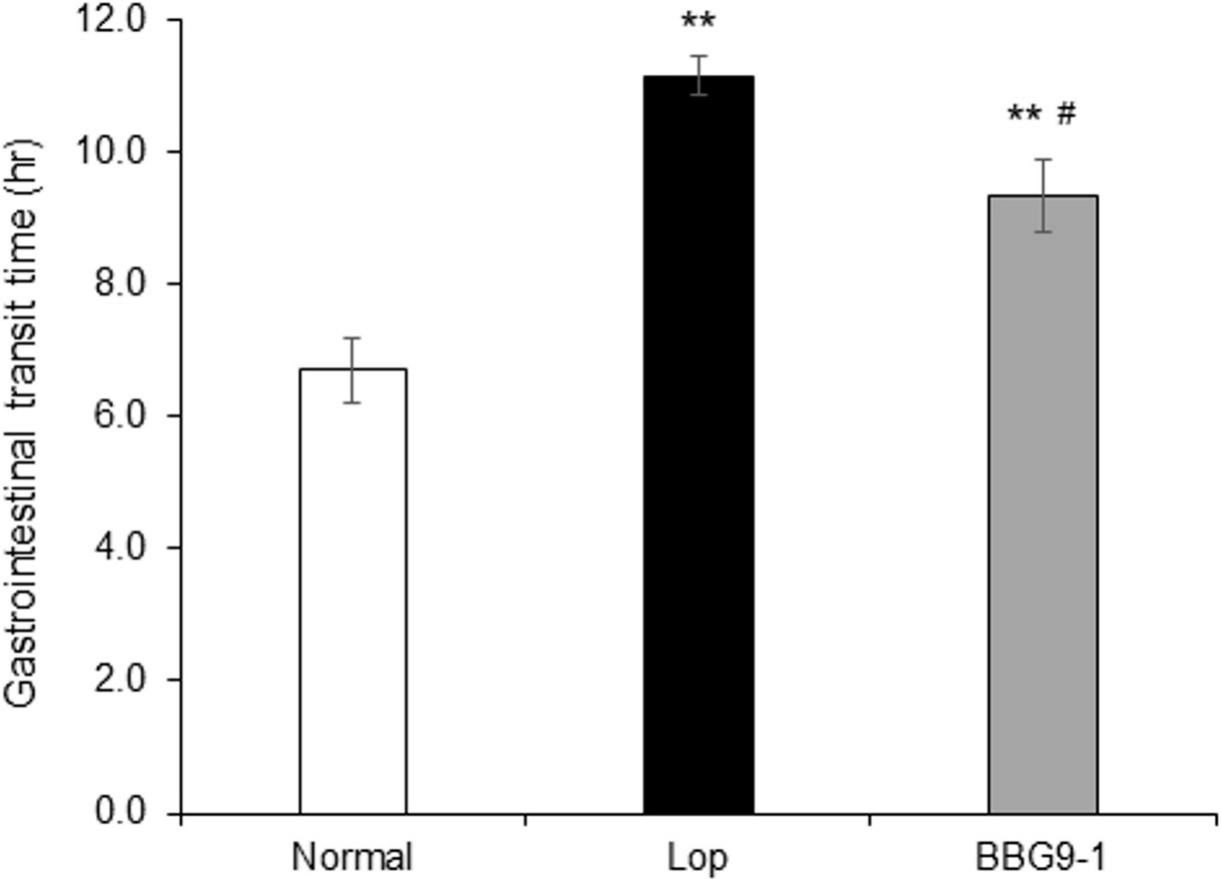
Influence of BBG9-1 on GITT. GITT was evaluated as the time during which the carmine dye was excreted as stool. Values are means ± SE of 10 animals. **, p < 0.01 vs. Normal; #, p < 0.05 vs. Lop.

Lop group showed significantly prolonged GITT compared with Normal group. BBG9-1 group also showed significantly prolonged GITT compared with the Normal group, but GITT was significantly shorter than that of the Lop group (Fig 3).

### Effect of BBG9-1 on loperamide-induced changes in fecal microbiota

**Fig 4:**
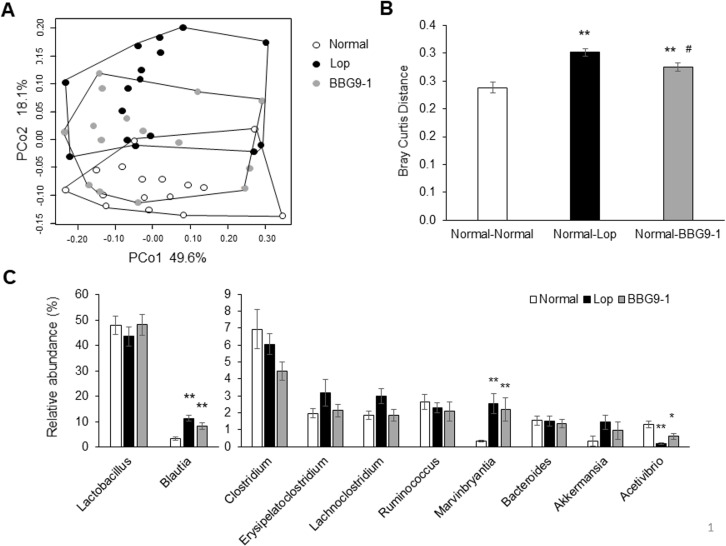
Effect of BBG9-1 on the structure of fecal microbiota in loperamide-induced constipation rats. (A) PCoA showed the clustered communities of fecal microbiota based on the Bray-Curtis dissimilarity between samples. (B) The calculated distance of Normal, Lop, and BBG9-1 groups in Bray-Curtis distance analysis. (C) The relative abundance of each bacterial genus was analyzed using 5000 next-generation sequencing reads of bacterial 16S rDNA. Values are means ± SE of 15 or 16 animals. * and **, p < 0.05 and 0.01 vs. Normal; #, p < 0.05 vs. Lop.

The PCoA plot based on the Bray-Curtis distance matrix revealed different fecal microbiota profiles between the Lop and Normal groups, indicating dysbiosis in loperamide-induced constipated animals. The fecal microbiota profile of the BBG9-1 group was located between Normal and Lop groups on the PCoA plot (Fig 4A). Bray-Curtis distances between samples from Normal and Lop groups and those between samples from the Normal and BBG9-1 group were significantly increased compared with distances between samples within the Normal group. Bray-Curtis distances between samples from Normal and BBG9-1 groups were significantly less than distances between samples from Normal and Lop groups (Fig 4B). When compared at the genus level, *Blautia* and *Marvinbryantia* were significantly more abundant in Lop and BBG9-1 groups than in the Normal group. In contrast, *Acetivibrio* was significantly less abundant in the Lop and BBG9-1 groups (Fig 4C).

### Effect of BBG9-1 on organic acids in cecal contents

Significant increases in acetic acid and propionic acid were observed in Lop and BBG9-1 groups. In contrast, butyric acid was significantly decreased in Lop group but not in BBG9-1 group animals, compared with the Normal group (Table 1).

**Table 1:** Effect of BBG9-1 on organic acids in cecal contents of loperamide-induced constipation model rat.

Treatment	Succinic acid (mg/g)	Lactic acid (mg/g)	Acetic acid (mg/g)	Propionic acid (mg/g)	n-Butyric acid (mg/g)	n-Valeric acid (mg/g)
Normal	0.27 ± 0.06	1.27 ± 0.25	3.72 ± 0.12	0.98 ± 0.04	3.44 ± 0.14	0.10 ± 0.01
Lop	0.25 ± 0.04	0.97 ± 0.21	4.51 ± 0.11 **	1.64 ± 0.07 **	2.69 ± 0.17 **	0.10 ± 0.01
BBG9-1	0.30 ± 0.06	1.29 ± 0.30	4.37 ± 0.18 **	1.49 ± 0.07 **	3.05 ± 0.16	0.10 ± 0.01

Values are means ± standard error (SE) of 16 animals. **p < 0.01, vs. Normal.

### Effect of BBG9-1 on serum 5-HT, DA, and Ach in loperamide-induced constipated rats

**Fig 5:**
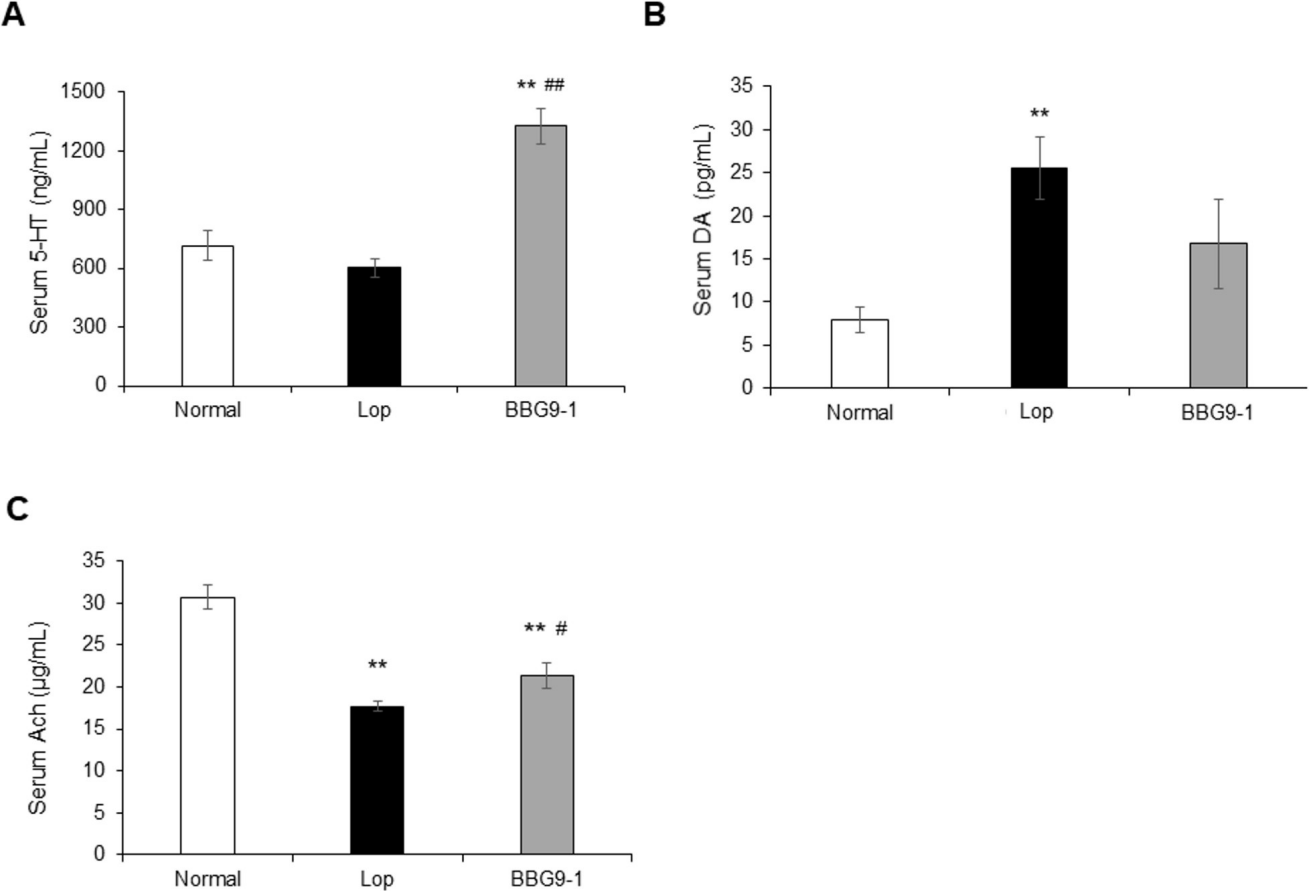
Effect of BBG9-1 on serum neurotransmitter in loperamide-induced constipation rats. The serum levels of (A) 5-HT and (B) DA were assayed with ELISA. The serum levels of (C) acetylcholine was assayed using Acetylcholine Assay Kit. Values are means ± SE of 9 or 10 animals. **, p < 0.01 vs. Normal; # and ##, p < 0.05 and 0.01 vs. Lop.

Serum 5-HT levels were not changed following loperamide treatment but were significantly increased in BBG9-1 group, compared with Normal and Lop groups (Fig 5A). Serum DA levels were significantly increased in Lop group compared with levels in Normal group. No significant differences were observed between BBG9-1 and Normal groups (Fig 5B). Serum Ach levels were significantly lower in both Lop and BBG9-1 groups than in the Normal group, but significantly higher in BBG9-1 group animals compared with rats in the Lop group (Fig 5C).

### Effect of BBG9-1 on Tph-1 expression in colon tissue of loperamide-induced constipated rats

**Fig 6:**
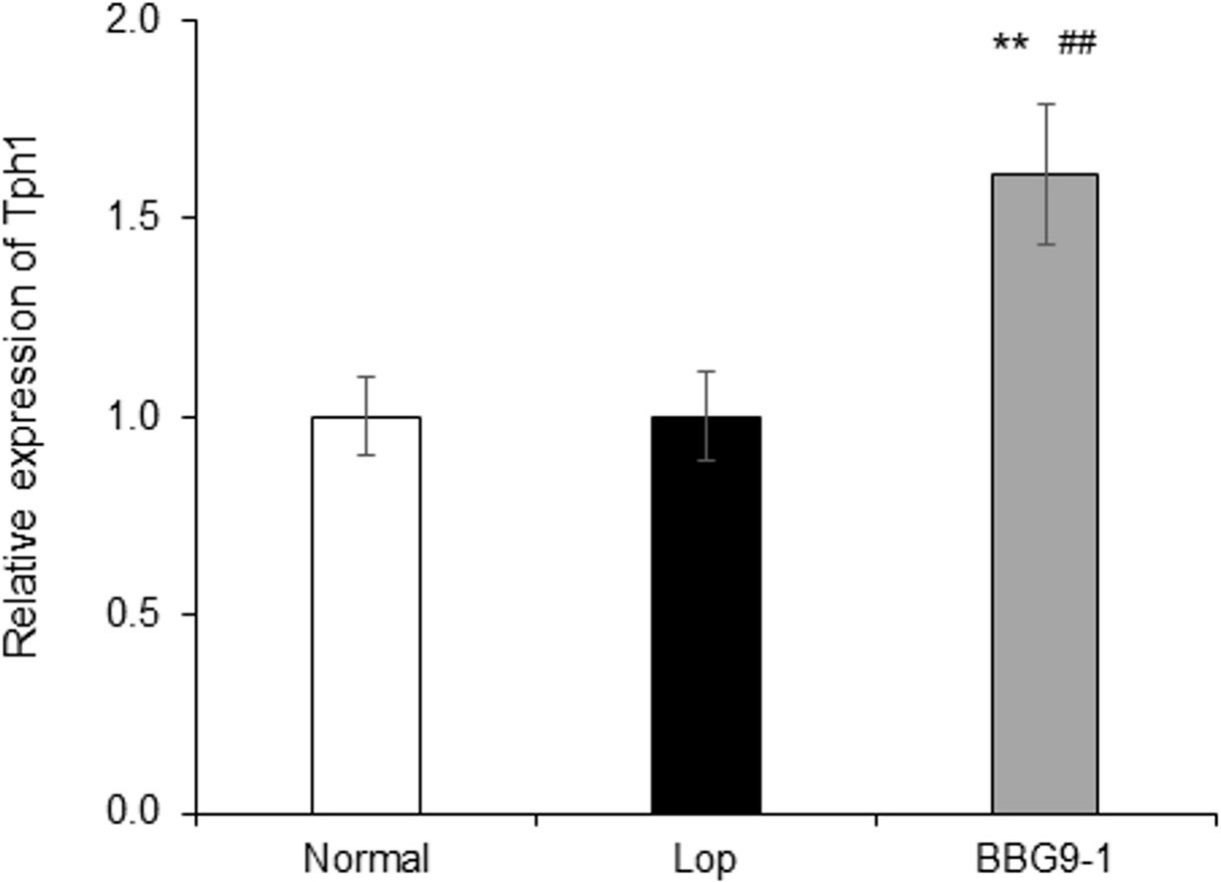
Relative expression of Tph-1 in distal colon of loperamide-induced constipation rats. Data were calculated using quantitative real-time PCR ΔΔCt method, and transcript expression was normalized using the housekeeping gene GAPDH. In the analysis, the relative gene expression level relative to the Normal group was calculated. Values are means ± SE of 9 or 10 animals. **, p < 0.01 vs. Normal; ##, p < 0.01 vs. Lop.

The expression level of Tph-1 in colon tissue was not different between the Lop and Normal groups but was significantly increased in the BBG9-1 group compared with Normal and Lop group animals (Fig 6).

## Discussion

In this study, we aimed to confirm that the probiotic BBG9-1 can improve constipation and to elucidate its mechanism of action. Loperamide induces STC-like constipation by inhibiting intestinal peristalsis and prolonging intestinal transit time [18, 19]. First, we investigated the effect of BBG9-1 on constipation using a rat model of loperamide-induced constipation. We and our colleagues have previously reported that BBG9-1 improves constipation as demonstrated by increased butyrate levels in the cecal contents and frequency of defecation in a rat model of low-fiber diet-induced constipation [20]. As expected, BBG9-1 improved fecal parameters in these animals. BBG9-1 also improved gut microbiota, prevented a reduction in butyric acid concentration in the gut, increased the expression of Tph-1, promoted 5-HT production, suppressed DA production, and enhanced Ach release.

Butyric acid, a metabolite of gut microbiota, promotes 5-HT production by increasing the expression of the 5-HT-producing enzyme, Tph-1 [13, 21–23]. A close relationship between 5-HT and gut microbiota is indicated by significantly decreased 5-HT levels in the serum and colon of GF mice compared to SPF mice [13], decreased 5-HT levels in constipated patients [12], and appearance of signs of constipation with decreased 5-HT levels in mice transplanted with feces from constipated patients [12]. 5-HT stimulates gastrointestinal motility by releasing acetylcholine through binding to specific receptors in peripheral nerves. These findings suggest that the trend of increasing butyric acid content in BBG9-1 group may contribute to a significant increase in serum Ach levels via upregulation of Tph-1 expression and serum 5-HT. These effects then lead to enhanced intestinal motility, shorter GITT and less accumulation of feces in the gut. DA inhibits Ach release by binding to its receptors, leading to a decrease in gastrointestinal motility. Gut dysbiosis is closely related to DA production [24]. Also, in this study, dysbiosis and a significant increase in DA were observed in Lop group, supporting the hypothesis that loperamide-induced dysbiosis contributes to DA production. Dysbiosis improved with BBG9-1 may therefore cause a reduction in DA production, leading to enhanced intestinal motility. Administration of DA significantly increases colonic water absorption in a colon loop model [24]. In addition to shortened intestinal transit time, decreased water absorption due to suppression of DA production, may contribute to increased fecal water content and reduced hardness observed with BBG9-1 administration observed in the present study.

One human study indicated that *Bifidobacteria* were not decreased in the gut microbiomes of the constipated patients, but butyrate-producing genera was low [25]. Butyrate could be one of main factors for the management of constipation. The administration of BBG9-1 previously showed an increase in the butyrate levels in rat cecal contents by improving the gut microbiota [20], although BBG9-1 did not directly produce butyrate. BBG9-1 produces lactic and acetic acids; however, in this study, concentrations of these acids were not significantly increased. Moreover, butyric acid was marginally increased in the BBG9-1 group, compared with animals in the Lop group. One explanation might be that BBG9-1 altered gut microbiota such that bacteria that produce butyric acid from lactic and acetic acids became more abundant. Butyrate-producing bacteria produce butyrate by consuming lactic acid and acetic acid [26, 27]. The observed increase in the content of intestinal butyrate may therefore be explained by the fact that lactic acid and acetic acid produced by BBG9-1 may be consumed by butyrate-producing bacteria, leading to an increase in the butyric acid content. Further study is warranted to demonstrate that the administration of butyric acid-producing bacteria improves constipation toward verifying that butyrate is a key factor. However, in this study, it was difficult to identify bacteria responsible for butyrate production, which may lead to the correction of the neurotransmitter profile as taxonomy analysis at the genus level did not identify bacteria that changed significantly after treatment. We plan to conduct a more detailed taxonomy analysis at the species or strain level, such as shotgun or long-read metagenomic sequencing, toward identify the key bacteria responsible for changes in butyrate production and neurotransmitter profiling and then to verify that the administration of the identified butyrate-producing bacteria improves constipation.

*Acetivibrio* produce H_2_ gas [28], which increases intestinal mobility [29]. Decreased *Acetivibrio* in the gut microbiota may contribute to the development of constipation. *Marvinbryantia* belong to the *Lachnospiraceae* family can produce acetate [30]. Increased *Marvinbryantia* may have contributed to the observed increase in the level of acetic acid in the Lop group. *Acetivibrio* were increased in the BBG9-1 group when compared with the Lop group, although the difference was not significant (calculated test statistic = -2.19782 and critical value at 0.05 level of significance = 2.343705). Therefore, BBG9-1 may increase the proportion of *Acetivibrio* in the gut microbiota, resulting in the improvement of constipation.

In this study, we attempted to elucidate the mechanism of action of BBG9-1 to improve constipation using a rat model. However, the intestinal microbiota differs between rats and humans. For example, *Lactobacillus* species are among the most abundant bacteria in rats but a minor component of human gut microbiota. The impact of host-differences such as these on the effect of BBG9-1 remains unknown. It is difficult to directly extrapolate results in this study to humans. Therefore, it is necessary to establish an animal model of constipation using germ-free mice transplanted with feces from constipated individuals for further understanding the mechanism of action of BBG9-1 in humans.

**Fig 7:**
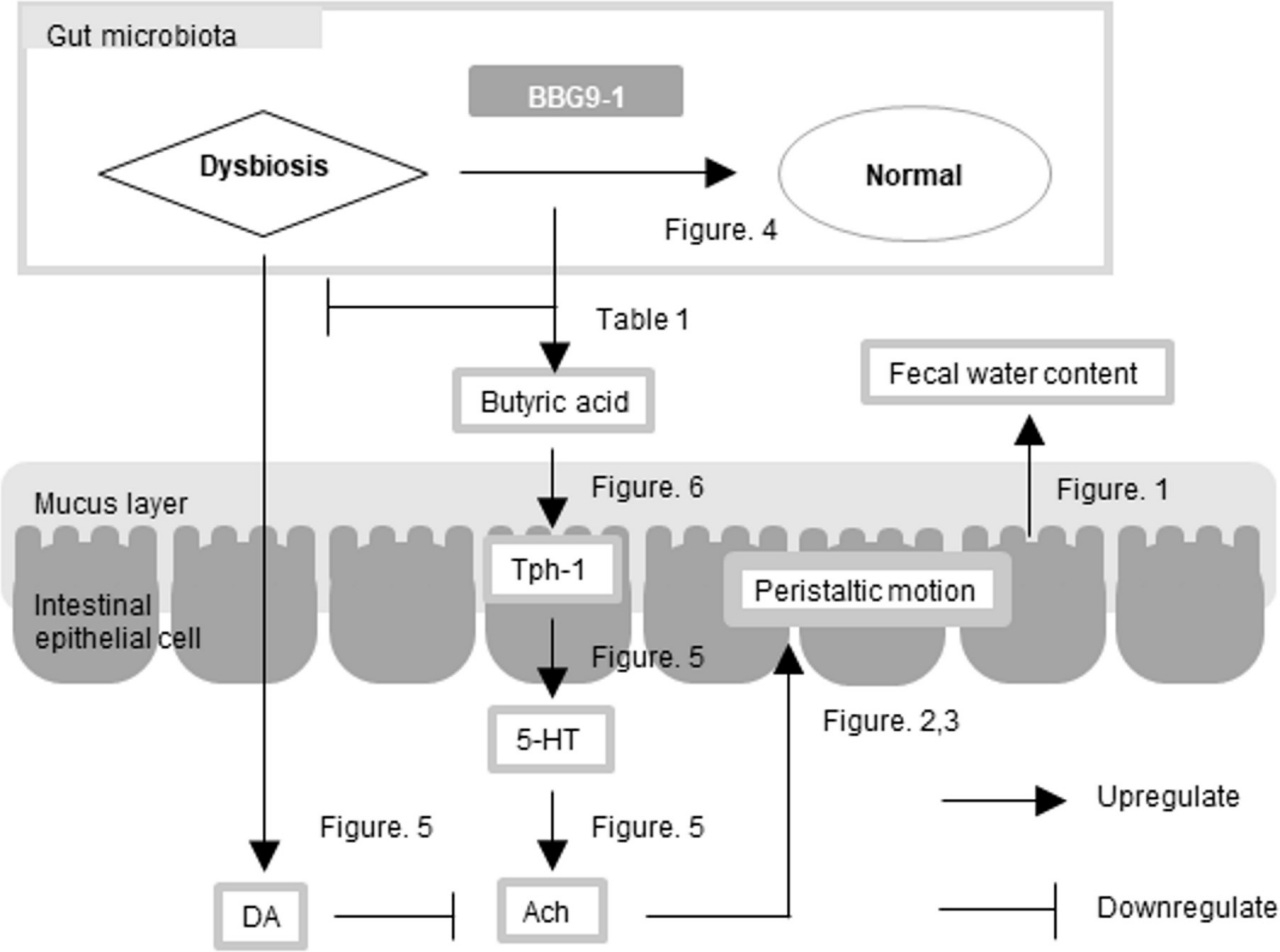
Outline of the influence of BBG9-1 on the loperamide-induced constipation rats. Loperamide-induced constipation was associated with dysbiosis. Correction of dysbiosis by BBG9-1 improved intestinal motility-related factors. As a result, peristalsis was enhanced and constipation symptoms were improved.

The results of this study indicate that the overall effect of BBG9-1 on loperamide-induced constipation in rats may be due to improved dysbiosis in gut microbiota and increased butyric acid-induced neurotransmitters-mediated intestinal motility (Fig 7). Oral administration of the probiotic BBG9-1 is thus a potential effective strategy for the management of constipation.

## Supporting information

S1 Dataset Research data set for this study. (XLSX)
