# Correction: Improvement of loperamide-induced slow transit constipation by *Bifidobacterium bifidum* G9-1 is mediated by the correction of butyrate production and neurotransmitter profile due to improvement in dysbiosis

**DOI:** 10.1371/journal.pone.0267927

**Published:** 2022-04-27

**Authors:** Yutaka Makizaki, Taiki Uemoto, Haruka Yokota, Miyuki Yamamoto, Yoshiki Tanaka, Hiroshi Ohno

In the Methods and Materials section, there is an error in the second sentence of the third paragraph. The correct sentence is: Rats in Lop and BBG9-1 groups were subcutaneously injected with 5.0 mg/kg of loperamide hydrochloride (Sigma, St. Louis, MO, USA), twice a daily, for 4 consecutive days to induce constipation.

There is an error in the caption for Fig 1. There are also errors in Fig 4. The authors have provided corrected versions of both below.

**Fig 1:**
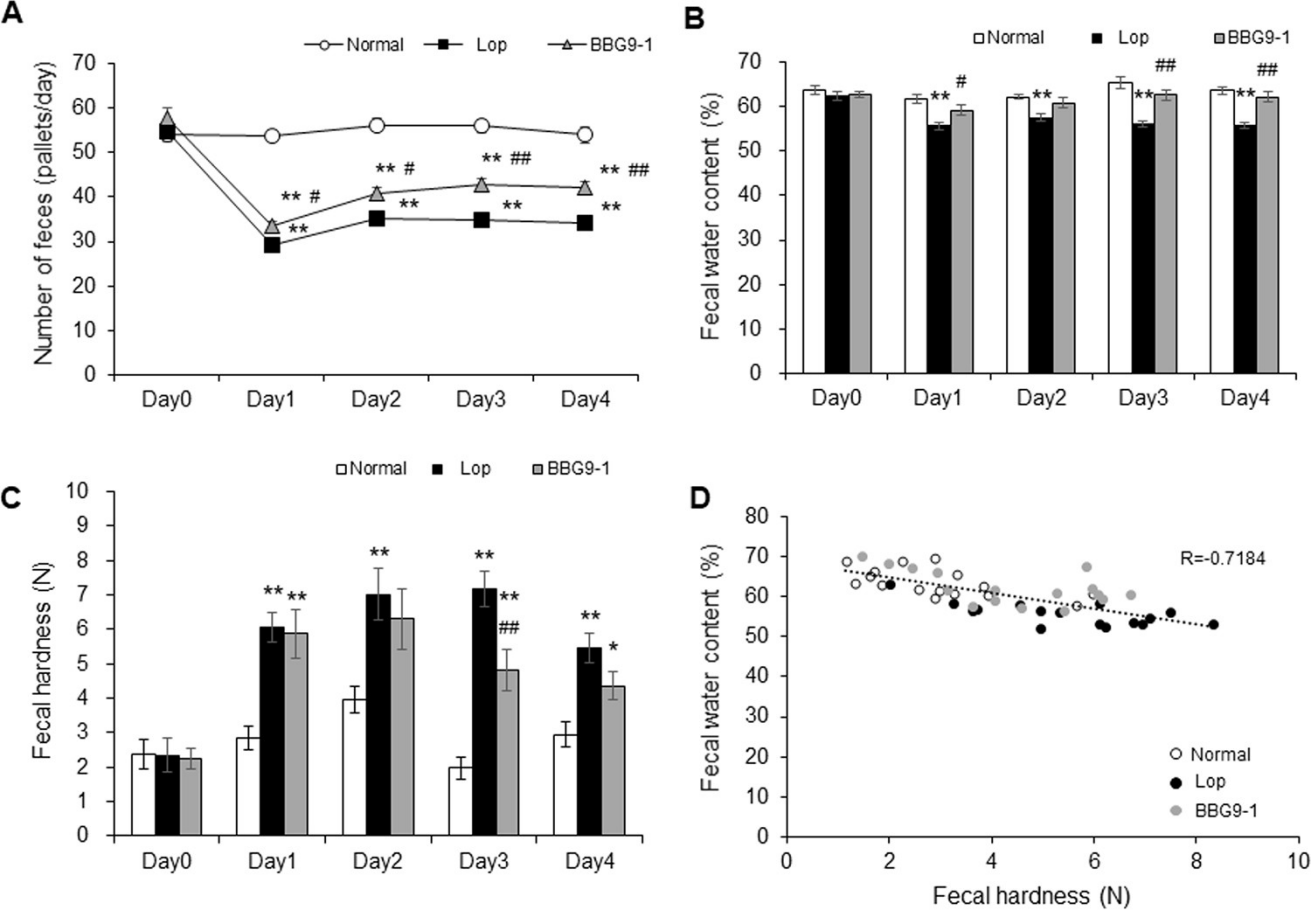
Change in fecal properties in rats with loperamide-induced constipation. (A) Number of feces, (B) Fecal water content, and (C) Fecal hardness were measured every experimental day. (D) Correlation between fecal water content and hardness was assessed at day 4. Values are means ± standard error (SE) of 16 animals. * and **, p < 0.05 and 0.01 vs. Normal; # and ##, p < 0.05 and 0.01 vs. Lop.

**Fig 4:**
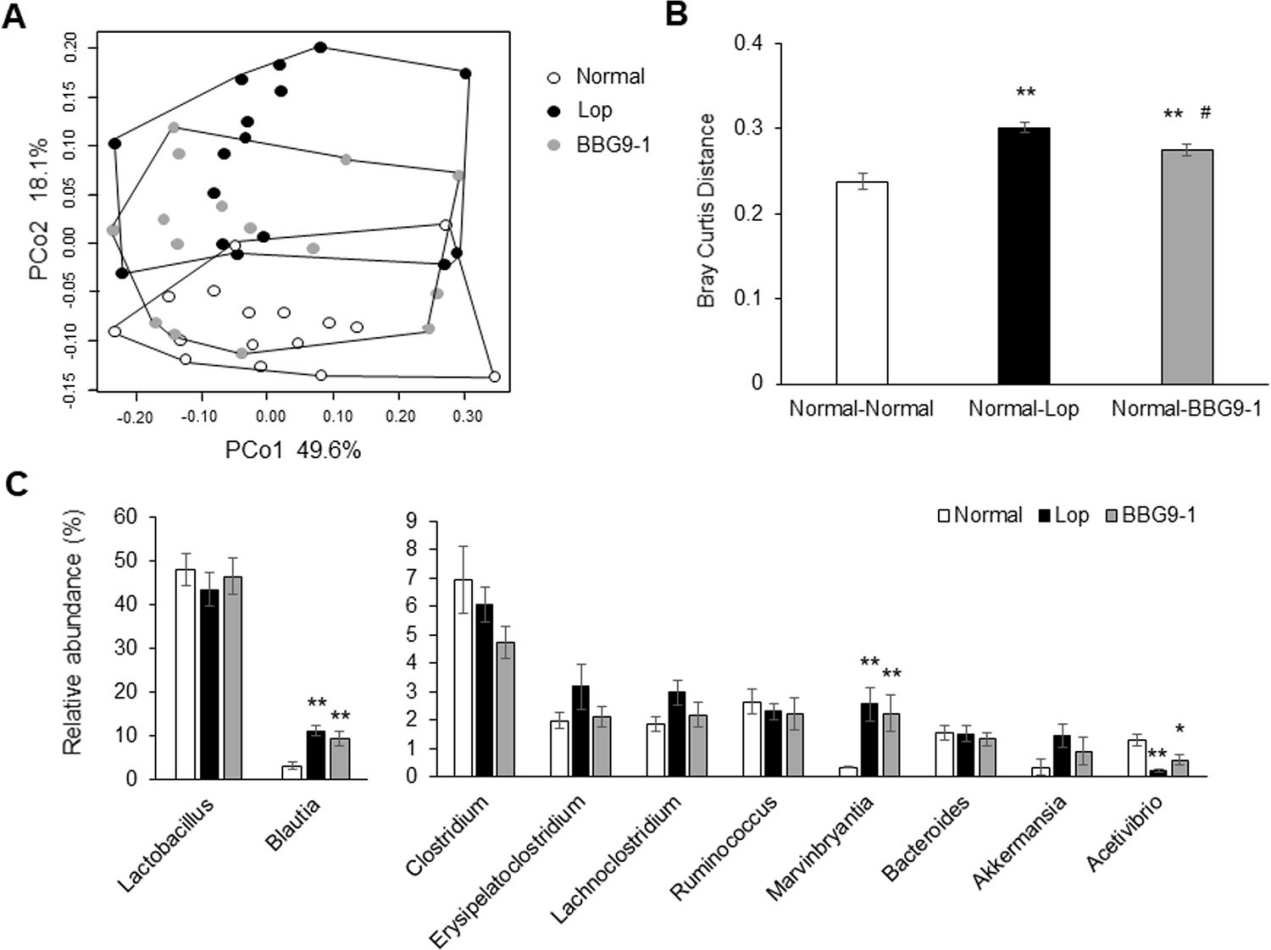
Effect of BBG9-1 on the structure of fecal microbiota in loperamide-induced constipation rats. (A) PCoA showed the clustered communities of fecal microbiota based on the Bray-Curtis dissimilarity between samples. (B) The calculated distance of Normal, Lop, and BBG9-1 groups in Bray-Curtis distance analysis. (C) The relative abundance of each bacterial genus was analyzed using 5000 next-generation sequencing reads of bacterial 16S rDNA. Values are means ± SE of 15 or 16 animals. * and **, p < 0.05 and 0.01 vs. Normal; #, p < 0.05 vs. Lop.
